# Sexual Dimorphism of Adipose and Hepatic Aquaglyceroporins in Health and Metabolic Disorders

**DOI:** 10.3389/fendo.2015.00171

**Published:** 2015-11-05

**Authors:** Amaia Rodríguez, Raul A. Marinelli, Angela Tesse, Gema Frühbeck, Giuseppe Calamita

**Affiliations:** ^1^Metabolic Research Laboratory, CIBEROBN, IdiSNA, Clínica Universidad de Navarra, Pamplona, Spain; ^2^Facultad de Ciencias Bioquímicas y Farmacéuticas, Instituto de Fisiología Experimental, Consejo Nacional de Investigaciones Científicas y Técnicas (CONICET), Universidad Nacional de Rosario, Rosario, Argentina; ^3^INSERM UMR 1087/CNRS UMR 629, L’Institut du Thorax, IRS-UN, Nantes, France; ^4^Department of Biosciences, Biotechnologies and Biopharmaceutics, University of Bari “Aldo Moro”, Bari, Italy

**Keywords:** glycerol, metabolism, aquaglyceroporins, obesity, insulin resistance, fatty liver disease, gender dimorphism

## Abstract

Gender differences in the relative risk of developing metabolic complications, such as insulin resistance or non-alcoholic fatty liver disease (NAFLD), have been reported. The deregulation of glycerol metabolism partly contributes to the onset of these metabolic diseases, since glycerol constitutes a key substrate for the synthesis of triacylglycerols (TAGs) as well as for hepatic gluconeogenesis. The present mini-review covers the sex-­related differences in glycerol metabolism and aquaglyceroporins (AQPs) and its impact in the control of adipose and hepatic fat accumulation as well as in whole-body glucose homeostasis. Plasma glycerol concentrations are increased in women compared to men probably due to the higher lipolytic rate and larger AQP7 amounts in visceral fat as well as the well-known sexual dimorphism in fat mass with women showing higher adiposity. AQP9 represents the primary route for glycerol uptake in hepatocytes, where glycerol is converted by the glycerol-kinase enzyme into glycerol-3-phosphate, a key substrate for *de novo* synthesis of glucose and TAG. In spite of showing similar hepatic AQP9 protein, women exhibit lower hepatocyte glycerol permeability than men, which might contribute to their lower prevalence of insulin resistance and NAFLD.

## Glycerol is an Important Variable in Metabolic and Energy Homeostasis

Glycerol represents a direct source of glycerol-3-phosphate (G3P), an important metabolite for the control of fat accumulation since it is required for the synthesis of triacylglycerols (TAGs), and for glucose homeostasis, given that it constitutes a major substrate for gluconeogenesis during states of negative energy balance, such as fasting or exercise (1, 2). Circulating free glycerol results from lipolysis, diet-derived glycerol, or glycerol reabsorbed in proximal renal tubules. In addition, intracellular glycerol also derives from glucose, via glycolysis, or through the conversion of pyruvate, lactate, and alanine to G3P, a pathway termed *glyceroneogenesis* occurring in the white and brown adipose tissues, and in the liver to support TAG synthesis, particularly in situations when cycling of TAG is increased (1).

Aquaglyceroporins (AQPs), protein channels allowing transport of glycerol, other small neutral solutes and water across membranes, are emerging as important players in metabolic and energy homeostasis with important implications in adiposity and insulin resistance control. In line with the proven preference of carbohydrate metabolism in men and lipid in women, important quantitative sexual-specific differences in AQPs and glycerol as metabolic substrate are being observed both in health and disease (3). Here, we will discuss the metabolic relevance and gender dimorphism of AQPs in fat and hepatic glycerol homeostasis with a particular focus on some worrisome metabolic disorders.

## Sexual Dimorphism of Aquaglyceroporin Expression and Glycerol Metabolism

### White Adipose Tissue and Liver are Central in Glycerol Homeostasis

During states of negative energy balance, such as fasting and exercise, adipocyte lipolysis is increased by the activation of adipose TAG lipase (ATGL) and hormone-sensitive lipase (HSL). Breakdown of TAG into free fatty acids (FFA) and monoacylglycerol (MAG) is initiated by ATGL and HSL, whereas the final step of converting MAG into FFA and glycerol is catalyzed by MAG lipase (4). Lipolysis occurs during low circulating insulin and under hormonal stimulation by catecholamines, through lipolytic β-adrenoceptors (β1, β2, and β3), and natriuretic peptides. Up to 65% of generated FFA is re-esterified back into TAG within white adipose tissue, whereas most of the generated glycerol is released from adipocytes to provide energy needs in other tissues, even in the fasted state. The activity of glycerol-kinase (GK), the enzyme catalyzing the initial phosphorylation of glycerol into G3P, in adipocytes is very low and the G3P used for TAG re-synthesis is mostly derived from the glyceroneogenesis (5).

**Figure 1:**
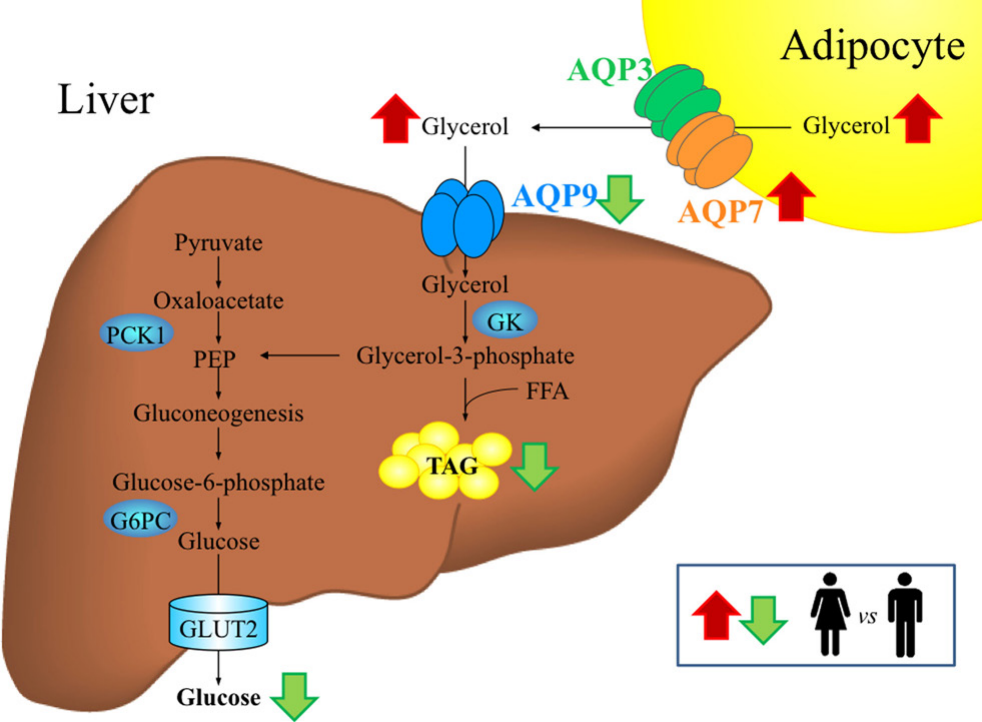
**Gender-specific differences in aquaglyceroporins and glycerol metabolism in adipose tissue and liver during fasting**. In cellular membranes, aquaporins assemble as a tetramer, with each monomer forming a functionally independent pore that allows the movement of water and/or other small solutes, such as glycerol, in the case of aquaglyceroporins. Women exhibit higher circulating glycerol concentrations than men during fasting, probably due to the higher percentage of fat mass characteristic of females as well as the higher lipolytic rate and AQP7 abundance in visceral adipose tissue. AQP9 represents the primary route for glycerol uptake in hepatocytes, where glycerol is converted to glycerol-3-phosphate by the GK enzyme for the *de novo* synthesis of glucose and triacylglycerols. Despite similar hepatic AQP9 expression, women exhibit lower hepatocyte glycerol permeability than men, which might contribute to the lower prevalence of NAFLD in women. The conditions showing upregulation (*red arrows*) or downregulation (*green arrows*) in women compared to men are indicated. FFA, free fatty acids, G6PC, glucose-6-phosphatase; GK, glycerol kinase; GLUT, glucose transporter; NAFLD, non-alcoholic fatty liver disease; PEP, phosphoenolpyruvate; PCK1, phosphoenolpyruvate carboxykinase variant 1; TAG, triacylglycerols.

The liver is responsible for 70–90% of the whole-body glycerol metabolism. Glycerol imported by hepatocytes is phosphorylated into G3P by GK enzyme. Depending on the metabolic state, G3P is used for lipogenesis (TAG) or gluconeogenesis, the latter process being the one using most of the generated G3P (1). G3P for TAG synthesis is also made from pyruvate by the enzymatic activity of pyruvate carboxylase (PC) and phosphoenolpyruvate carboxykinase (PEPCK), an enzyme whose activity is reduced by insulin and other hormones (Figure 1). The proportion of glycerol that ends up in TAG synthesis vs. gluconeogenesis is correlated with a number of parameters whose actions mostly convey gender-based differences (3). In general, the extent of G3P used for gluconeogenesis increases as the fasting period lasts.

### Gender-Specific Differences in Glycerol Metabolism

Hepatic glycerol utilization involves sexual dimorphism, a trait reflecting the preference of carbohydrate metabolism in men and lipid in women. In healthy humans, important gender-specific differences in glycerol metabolism exist in states of increased energy demand, such as hypoglycemia, fasting, or exercise (3).

During hypoglycemia, despite the higher neuroendocrine and autonomic nervous system (ANS) responses exhibited by females compared to males (6), females have higher plasma glycerol compared to males (Figure 1). This could be explained by a greater rate in glycerol turnover resulting from increased peripheral fat accumulation and higher percent of fat mass characterizing females. Moreover, female adipose tissue shows higher sensitivity to lipolytic hormones, such as epinephrine, norepinephrine, and growth hormone, compared to men (7). Greater plasma glycerol in women is also attributed to the higher lipolysis and fat oxidation exhibited by women during hypoglycemia compared to men.

After 72-h fasting, correlating with the sex-based differences in plasma glucose and FFA, women showed significantly higher plasma glycerol levels compared to men (8). Changes in glycerol metabolism between genders may relate to different sensitivities to insulin and epinephrine, two hormones inhibiting and stimulating lipolysis, respectively, and leptin, a hormone with lipolytic effects (3, 4, 9). Obesity is associated with higher lipolytic rates leading to increased circulating FFA and glycerol (10); however, the sex-specific relevance of these hormones in glycerol homeostasis remains to be fully assessed.

Gender-related differences in lipolytic glycerol utilization also exist during physical exercise. After 90 min of moderate exercise, women have significantly higher levels of plasma glycerol as compared to men, a counterregulatory response consistent with the reduced carbohydrate and increased lipid utilization characterizing women (6). Changes in ANS responses have been suggested to be the major factor responsible of this metabolic finding (3). No effect of the cyclic fluctuations in estrogen and progesterone occurring during the menstrual cycle was observed as regards the systemic rates of glycerol during 90 min of moderate exercise (11).

### Aquaglyceroporins, Metabolic Gateways Facilitating the Transport of Glycerol Across the Cell Membrane in Adipose Tissue and Liver

Aquaglyceroporins (AQP3, 7, 9, and 10), a subgroup of the aquaporin family of channel proteins allowing transport of glycerol and some other neutral solutes besides to water across biological membranes, have emerged as important metabolic gateways in adiposity and insulin resistance control (12–14).

AQP7 represents the main pathway in facilitating release of lipolytic glycerol from adipocytes (15–21), although some authors have not been able to detect AQP7 expression in fat cells (22). Nonetheless, other glycerol channels, such as AQP3, AQP10, and the more recently identified AQP11, contribute to glycerol efflux from fat, but to a lower extent (19, 20, 23). Adipocytes also express AQP9, an AQP believed to mediate the entry of glycerol into fat tissue (19), and AQP5, a water channel involved in the process of adipocyte differentiation (24). The expression of AQPs shows fat depot differences with visceral fat exhibiting higher expression of AQP3 and AQP7, which might reflect an overall increase in lipolytic rate and glycerol release in this fat depot, and subcutaneous fat showing lower AQP7 levels pointing to the promotion of an intracellular glycerol accumulation and a progressive adipocyte hypertrophy (17, 19, 25, 26).

Glycerol released by adipocytes, via the bloodstream, is imported by hepatocytes through AQP9, the principal facilitative pathway in liver uptake of glycerol (27, 28) localized at the sinusoidal domain of the cell membrane, facing the spaces of Disse (29). In the rodent liver, AQP9 displays a heterogeneous expression pattern. Especially in females, hepatocyte AQP9 protein expression is greater in the area surrounding the central vein by gradually declining toward the periportal area. Sexual dimorphism is also indicated by female rats having significantly lower levels of hepatic AQP9 protein compared with males in both fed and fasting conditions (30, 31). In addition to AQP9, although at low levels, human hepatocytes also express three other AQPs, AQP3, AQP7, and AQP10 (10). The sinusoidal uptake of glycerol is of critical importance as glycerol utilization by the liver is rate limited at the membrane permeation step (32). GK catalyzes the initial step for the conversion of the imported glycerol into ­glycerol-3-phosphate, an important substrate for *de novo* synthesis of glucose (gluconeogenesis) and/or TAG (lipogenesis) (Figure 1).

### Hormonal Regulation and Sexual Dimorphism of Fat and Liver Aquaglyceroporins

The coordinated regulation of AQPs in adipocytes and hepatocytes is pivotal in maintaining the control of fat accumulation in adipose tissue and liver, as well as whole-body glucose homeostasis. In this sense, *Aqp7* gene disruption leads to obesity (33, 34), and *Aqp9* deficiency is related to a defective hepatic glycerol metabolism in mice (2). However, the interaction with the genetic background of the transgenic mice as well as the age of the experimental animals appears to influence these metabolic phenotypes. In this regard, *Aqp7*-deficient mice backcrossed into the C57BL/6N genetic background develop adult-onset obesity (33, 35) but not in the young state (22, 36), whereas *Aqp7*-knockout mice generated in CD1 mice did not exhibit excess body weight but showed an increase in whole-body fat content (34). In the same line, *Aqp9* deletion in leptin receptor-deficient *db/db* mice in a mixed C57BL/6 × C57BLKS genetic background reduces postprandial blood glucose levels (2), whereas in *Aqp9-*deficient C57BL/6 *db/db* mice elevates plasma glucose and does not alleviate hepatosteatosis (37).

**Figure 2:**
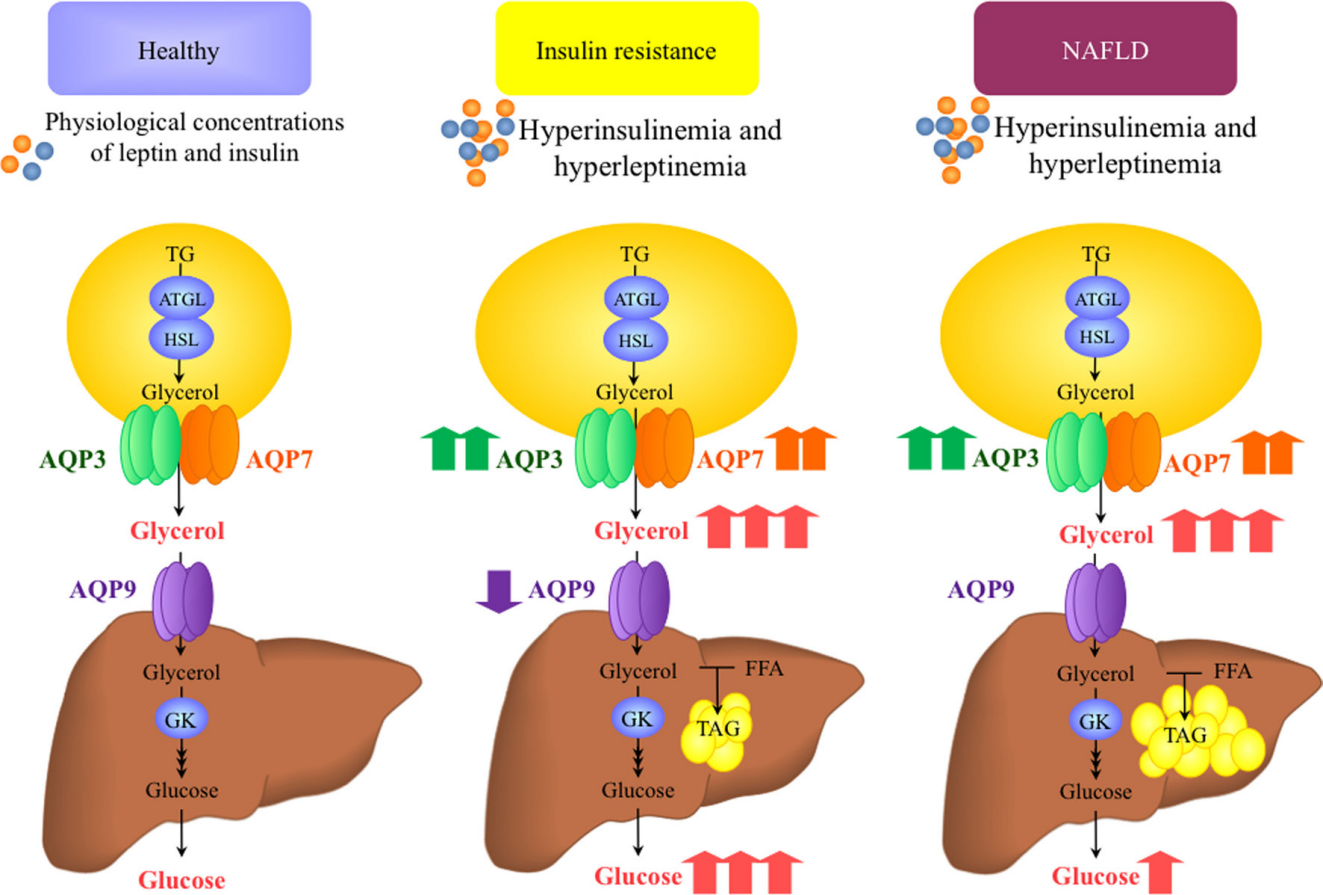
**Proposed working model for the role of aquaglyceroporins in the onset of insulin resistance and non-alcoholic fatty liver disease (NAFLD)**. Insulin and leptin are regulatory factors for the expression of AQP3 and AQP7 glycerol channels in adipocytes and AQP9 in hepatocytes. Plasma insulin and leptin concentrations change in accordance to the nutritional state and adiposity, respectively, and therefore, the expression of aquaglyceroporins in adipose tissue and liver increases or decreases in relation to the nutritional needs and excess fat mass. In the setting of obesity-associated insulin resistance and NAFLD, AQP3 and AQP7 are overexpressed in the adipose tissue, despite the hyperleptinemia. Consequently, glycerol output from fat cells and glycerol use for hepatic gluconeogenesis and lipogenesis increase. The reduced AQP9 expression and glycerol permeability in the liver of obese patients with insulin resistance seems to be a defensive mechanism to prevent a further increase in hepatic steatosis and hyperglycemia. ATGL, adipose tissue triacylglycerol lipase; HSL, hormone-sensitive lipase; FFA, free fatty acids; GK, glycerol kinase; TAG, triacylglycerol.

In rodents, insulin represses adipose *Aqp7* and hepatic *Aqp9* gene expression through the negative insulin response element (IRE) in their gene promoters (15, 38). Rodent models of insulin resistance, such as streptozotocin-induced diabetic rats or genetically obese *db/db* mice, have an increase in hepatic AQP9 (38, 39). Gender-specific differences have been described in hepatic AQP9 modulation by insulin in rodents. During fasting, hepatic AQP9 protein increased 2.6-fold in male compared to female rats (31). Coordinately, plasma glycerol levels remained unchanged with starvation in male rats, whereas they were increased in female rats. Consistent with the major role played by AQP9 in rodent liver glycerol import, the different responses to starvation were paralleled by higher hepatic glycerol permeability in starved male rats compared with starved females. Interestingly, ovariectomy led to a starvation-response pattern identical to that observed in male rats with increased hepatic AQP9 expression and unchanged plasma glycerol levels. These results, together with those obtained with cultured hepatocytes challenged with 17β-estradiol and an estrogen receptor β-agonist, led to the hypothesis that the sex-specific regulation of AQP9 during starvation contributes to the higher plasma glycerol levels characterizing females during fasting (31). A recent work showed that the mRNA expression of *Aqp9* in male rat periportal hepatocytes was reduced in response to the peroxisome proliferator-activated receptor α (PPARα) (40). Contrary to what is observed in rodents, insulin upregulates the expression of AQPs in human HepG2 hepatocytes, a control that occurs through the activation of the PI3K/Akt/mTOR pathway (19). In liver sections of obese patients, AQP3 and AQP7 show a cytoplasmatic distribution surrounding lipid droplets (19), while a strong immunoreactivity of AQP9 appears in the basolateral membrane of the hepatocytes (10, 19). Thus, insulin-mediated elevation of AQP3 and AQP7 may reflect an increase in intrahepatocellular TAG content induced by the hormone in HepG2 hepatic cell line, whereas insulin-induced AQP9 upregulation seems to facilitate glycerol import by hepatocytes. This may explain why obese patients with type 2 diabetes (T2D) have decreased hepatic AQP9, compatible with a compensatory mechanism aimed at reducing the glycerol entry into hepatocytes and further enhancing the development of hyperglycemia (Figure 2) (10, 19). The mRNA expression of *AQP9* in HepG2 cells was modulated by the AMP-activated protein kinase (AMPK), a known energy sensor in cells, via forkhead box a2 (41). Under physiological conditions, AMPK activation occurs in response to an increase in the intracellular AMP/ATP ratio. Connection between obesity and secondary development of T2D and a common SNP (A953G) in the *AQP7* promoter was reported in Caucasian individuals. *AQP7* gene transcription was decreased due to the impaired C/EBPβ binding to the promoter triggered by the polymorphism (42). The unique reported rare case of human *AQP7* deficiency was not associated with the onset of obesity and diabetes as occurs in *Aqp7-*knockout animals (43). Interestingly, this *AQP7*-deficient individual lacked exercise-induced increase in adipose tissue glycerol release in spite of elevated plasma levels of the lipolytic hormone noradrenaline. Interestingly, a gender-specific effect of physical training on adipose AQP7 expression has been reported with women showing higher abundance of AQP7 compared with men (44) and increasing the abundance of AQP7 in abdominal subcutaneous fat depot after a 10-week endurance exercise program (45).

Leptin, an adipocyte-derived hormone with lipolytic action, has been shown to repress the AQP7 and AQP9 protein expression via the PI3K/Akt/mTOR signaling cascade in differentiated human adipocytes and hepatocytes, respectively (19). The lipolytic stimulation with leptin and catecholamines induce the translocation of AQP7 from the cytoplasm, surrounding lipid droplets, to the plasma membrane, favoring glycerol release from adipocytes (19, 46, 47). Nonetheless, the direct stimulation of adipocytes with catecholamines (19, 48) or leptin (19, 47) downregulates the mRNA and protein expression of AQP7 in adipocytes, suggesting a negative feedback regulation of AQP7 in lipolytic states to restrict glycerol release from adipocytes in order to prevent the depletion of fat stores. The sexual dimorphism in leptin regulation of human adipose and hepatic AQPs warrants investigation as women have two to three times higher levels of circulating leptin compared with men (49). Leptin showed a distinct mechanism of action toward rodent AQPs. Acute leptin treatment induced the translocation of AQP3 and AQP7 to lipid droplets and the plasma membrane of murine adipocytes, respectively, and facilitation of glycerol mobilization after lipolysis was therefore reasonably suggested (47). Chronic leptin administration in male leptin-deficient *ob/ob* mice down-regulated adipose AQP3 and AQP7 at the same time as it upregulated hepatic AQP9 in parallel with the improvement of obesity and hepatosteatosis observed in this genetically obese animal model (47). Positive correlation between PPARγ and adipose AQP3 and AQP7 and hepatic AQP9 was found regarding the regulatory effect exerted by leptin on mouse AQPs (47).

## AQP9 and Altered Hepatocyte Import of Glycerol, A New Intersecting Component in NAFLD/NASH

Non-alcoholic fatty liver disease (NAFLD) is characterized by ectopic accumulation of TAG in hepatocytes in response to metabolic, toxic, and viral insults (50), and it constitutes the leading cause of chronic liver disease with an estimated prevalence of 20–40% in Western countries (51). NAFLD may evolve to the inflammatory-fibrogenic form, the non-alcoholic steatohepatitis (NASH), the most worrisome form carrying a higher risk of developing liver cirrhosis, and hepatocellular carcinoma (52). A gender influence does exist in NAFLD/NASH with men being more commonly affected than women (53).

Multiple studies have been recently focusing on the pathways leading to the pathological accumulation of TAG in hepatocytes in NAFLD/NASH. Dysregulated hepatic FFA export, oxidation and desaturation, as well as systemic and hepatic insulin resistance are among the main pathways in NAFLD pathogenesis (54, 55). Altered glycerol uptake by hepatocytes is also a major intersecting component; however, the underlying mechanism has begun to be understood only recently, after proving the role played by AQPs in facilitating the transmembrane transport of glycerol in adipose tissue and liver (56).

A reduction of hepatocyte AQP9 protein with no changes in mRNA level was observed in n3-PUFA (ω3 polyunsaturated fatty acids)-depleted female rats, a model of metabolic syndrome displaying several features of the disease also including hepatosteatosis (57). Increased liver glycerol uptake was observed despite the AQP9 downregulation, an apparent discrepancy attributed to a raise in GK activity resulting from the altered intracellular metabolism. Pathophysiological involvement of AQP9 in NAFLD was also found in a study using male rats fed a high-fat diet (HFD) where a considerable reduction of the HFD-induced steatosis was observed after knocking down liver *Aqp9* (54). Our study with *ob/ob* mice, an animal model of NAFLD, showed decreased hepatocyte AQP9 and glycerol permeability correlated with increased plasma glycerol, with leptin administration restoring hepatic AQP9 levels and hepatosteatosis (47). Moreover, surgically induced weight loss is also associated with an improvement of the fatty liver in obese rats through the restoration of the coordinated regulation of AQPs in the adipose tissue and liver (26).

Hepatic *AQP9* mRNA was negatively correlated with intrahepatic lipid content in obese patients with hepatosteatosis (10, 17, 19). However, another study did not find any relationship between *AQP9* expression and the degree of hepatic steatosis or fibrosis in patients with morbid obesity (58). Recently, we used liver biopsies obtained from a mixed gender cohort of morbid obese patients undergoing bariatric surgery showing decreased hepatic expression of AQP9 in obese subjects with NAFLD (Figure 2) (10). Importantly, the AQP9 diminution paralleled the degree of hepatic steatosis, an observation that corroborated the notion that lower intrahepatocellular glycerol due to a decreased AQP9 expression may represent a compensatory mechanism to reduce the *de novo* TAG synthesis in fatty hepatocytes.

## Gender-Specific Differences in Hepatic Handling of Glycerol in Morbid Obese Patients with NAFLD

### Obese Women Have Lower Hepatic Glycerol Permeability Compared to Obese Men

Sexual dimorphism in hepatic import of glycerol in fat overaccumulation was found in a recent work by our group (10). A gender separated analysis of hepatic AQP9 regulation was performed in obese subjects with NAFLD undergoing bariatric surgery. Interestingly, obese women showed significantly lower hepatic glycerol permeability when compared with obese men (10). Consistent with a previous work by another group (58), the hepatic expression of AQP9 was similar between the two genders. All women used for this study were in a premenopausal state and females during pregnancy or lactation period were excluded, suggesting that the hepatic suppression of AQP9 by estrogens seen in female rats during short-term fasting (31) may not apply to humans, at least in morbid obese women with NAFLD. Why obese male and female patients with NAFLD have comparable hepatic expression of AQP9 but different glycerol permeability is not known, therefore, warranting investigation. Also, whether this disjunction is a general trait of humans reflecting gender-specific differences in energy-substrate utilization patterns, or it is restricted to morbid obese subjects remains elusive. Human hepatocytes also express three other AQPs, such as AQP3, AQP7, and AQP10, but to a lower extent than AQP9 (10) and fat overaccumulation might involve sex-specific regulation of these glycerol channels turning in higher hepatic glycerol permeability in male than female subjects. However, this may not be the case for AQP3 since this AQP was down-regulated in HepG2 cells with oleic acid-induced steatosis (59). Sexual-specific changes in the lipids composing the hepatocyte plasma membrane during hepatosteatosis might constitute another potential explanation. The lipid bilayer composing the sinusoidal plasma membrane provides an important contribution to the transport of glycerol into hepatocytes as proved in our recent work where nearly 50% of the glycerol flowing into murine hepatocytes during short-term fasting moved by the simple diffusion through the membrane lipid bilayer (27).

## Conclusion and Perspectives

Taken together, growing evidence points to a potential contribu­tion of glycerol metabolism and AQPs to the lower prevalence of insulin resistance in premenopausal women (Figure 2). The mechanisms underlying these gender-specific differences in humans include (i) sexual dimorphism in fat distribution, (ii) higher lipolysis and AQP7 in visceral fat, and (iii) lower hepatic glycerol permeability despite similar expression of AQP9 in women. Although rodents do not always represent reliable models to assess the relevance of aquaporins in human metabolism and energy balance, further investigations pertaining the hormonal regulation of AQPs in other metabolic tissues, such as skeletal muscle, intestine, pancreas, or heart, will shed more light on additional and unexpected metabolic roles of these molecules in the coming years.

## Conflict of Interest Statement

The authors declare that the research was conducted in the absence of any commercial or financial relationships that could be construed as a potential conflict of interest.

## References

[1] Reshef L Olswang Y Cassuto H Blum B Croniger CM Kalhan SC Glyceroneogenesis and the triglyceride/fatty acid cycle. J Biol Chem (2003) 278:30413–6. 10.1074/jbc.R300017200 12788931

[2] Rojek AM Skowronski MT Fuchtbauer EM Fuchtbauer AC Fenton RA Agre P Defective glycerol metabolism in aquaporin 9 (AQP9) knockout mice. Proc Natl Acad Sci U S A (2007) 104:3609–14. 10.1073/pnas.0610894104 17360690 PMC1805577

[3] Hedrington MS Davis SN. Sexual dimorphism in glucose and lipid metabolism during fasting, hypoglycemia, and exercise. Front Endocrinol (2015) 6:61. 10.3389/fendo.2015.00061 25964778 PMC4410598

[4] Frühbeck G Méndez-Gimenez L Fernández-Formoso JA Fernández S Rodríguez A. Regulation of adipocyte lipolysis. Nutr Res Rev (2014) 27:63–93. 10.1017/S095442241400002X 24872083

[5] Leroyer SN Tordjman J Chauvet G Quette J Chapron C Forest C Rosiglitazone controls fatty acid cycling in human adipose tissue by means of glyceroneogenesis and glycerol phosphorylation. J Biol Chem (2006) 281:13141–9. 10.1074/jbc.M512943200 16524879

[6] Davis SN Shavers C Costa F. Differential gender responses to hypoglycemia are due to alterations in CNS drive and not glycemic thresholds. Am J Physiol Endocrinol Metab (2000) 279:E1054–63. 11052960 10.1152/ajpendo.2000.279.5.E1054

[7] Schmidt SL Bessesen DH Stotz S Peelor FF III Miller BF Horton TJ. Adrenergic control of lipolysis in women compared with men. J Appl Physiol (1985) (2014) 117:1008–19. 10.1152/japplphysiol.00003.2014 25190743 PMC4217046

[8] Clore JN Glickman PS Helm ST Nestler JE Blackard WG. Accelerated decline in hepatic glucose production during fasting in normal women compared with men. Metabolism (1989) 38:1103–7. 10.1016/0026-0495(89)90047-4 2811679

[9] Frühbeck G Gómez-Ambrosi J Salvador J. Leptin-induced lipolysis opposes the tonic inhibition of endogenous adenosine in white adipocytes. FASEB J (2001) 15:333–40. 10.1096/fj.00-0249com 11156949

[10] Rodríguez A Gena P Méndez-Gimenez L Rosito A Valentí V Rotellar F Reduced hepatic aquaporin-9 and glycerol permeability are related to insulin resistance in non-alcoholic fatty liver disease. Int J Obes (2014) 38:1213–20. 10.1038/ijo.2013.234 24418844

[11] Horton TJ Miller EK Bourret K. No effect of menstrual cycle phase on glycerol or palmitate kinetics during 90 min of moderate exercise. J Appl Physiol (1985) (2006) 100:917–25. 10.1152/japplphysiol.00491.2005 16467391

[12] Frühbeck G Obesity: aquaporin enters the picture. Nature (2005) 438:436–7. 10.1038/438436b 16306977

[13] Rodríguez A Catalán V Gómez-Ambrosi J Frühbeck G. Aquaglyceroporins serve as metabolic gateways in adiposity and insulin resistance control. Cell Cycle (2011) 10:1548–56. 10.4161/cc.10.10.15672 21502813 PMC3127157

[14] Lebeck J. Metabolic impact of the glycerol channels AQP7 and AQP9 in adipose tissue and liver. J Mol Endocrinol (2014) 52:R165–78. 10.1530/JME-13-0268 24463099

[15] Kishida K Shimomura I Nishizawa H Maeda N Kuriyama H Kondo H Enhancement of the aquaporin adipose gene expression by a peroxisome proliferator-activated receptor γ. J Biol Chem (2001) 276:48572–9. 10.1074/jbc.M103555200 11679588

[16] Marrades MP Milagro FI Martínez JA Moreno-Aliaga MJ. Differential expression of aquaporin 7 in adipose tissue of lean and obese high fat consumers. Biochem Biophys Res Commun (2006) 339:785–9. 10.1016/j.bbrc.2005.11.080 16325777

[17] Catalán V Gómez-Ambrosi J Pastor C Rotellar F Silva C Rodríguez A Influence of morbid obesity and insulin resistance on gene expression levels of AQP7 in visceral adipose tissue and AQP9 in liver. Obes Surg (2008) 18:695–701. 10.1007/s11695-008-9453-7 18401671

[18] Miranda M Escote X Ceperuelo-Mallafre V Alcaide MJ Simon I Vilarrasa N Paired subcutaneous and visceral adipose tissue aquaporin-7 expression in human obesity and type 2 diabetes: differences and similarities between depots. J Clin Endocrinol Metab (2010) 95:3470–9. 10.1210/jc.2009-2655 20463097

[19] Rodríguez A Catalán V Gómez-Ambrosi J García-Navarro S Rotellar F Valentí V Insulin- and leptin-mediated control of aquaglyceroporins in human adipocytes and hepatocytes is mediated via the PI3K/Akt/mTOR signaling cascade. J Clin Endocrinol Metab (2011) 96:E586–97. 10.1210/jc.2010-1408 21289260

[20] Laforenza U Scaffino MF Gastaldi G. Aquaporin-10 represents an alternative pathway for glycerol efflux from human adipocytes. PLoS One (2013) 8:e54474. 10.1371/journal.pone.0054474 23382902 PMC3558521

[21] Miyauchi T Yamamoto H Abe Y Yoshida GJ Rojek A Sohara E Dynamic subcellular localization of aquaporin-7 in white adipocytes. FEBS Lett (2015) 589:608–14. 10.1016/j.febslet.2015.01.025 25643985

[22] Skowronski MT Lebeck J Rojek A Praetorius J Fuchtbauer EM Frokiaer J AQP7 is localized in capillaries of adipose tissue, cardiac and striated muscle: implications in glycerol metabolism. Am J Physiol Renal Physiol (2007) 292:F956–65. 10.1152/ajprenal.00314.2006 17077387

[23] Madeira A Fernández-Veledo S Camps M Zorzano A Moura TF Ceperuelo-Mallafré V Human aquaporin-11 is a water and glycerol channel and localizes in the vicinity of lipid droplets in human adipocytes. Obesity (2014) 22:2010–7. 10.1002/oby.20792 24845055

[24] Madeira A Mosca AF Moura TF Soveral G. Aquaporin-5 is expressed in adipocytes with implications in adipose differentiation. IUBMB Life (2015) 67:54–60. 10.1002/iub.1345 25631586

[25] Ceperuelo-Mallafré V Miranda M Chacón MR Vilarrasa N Megia A Gutiérrez C Adipose tissue expression of the glycerol channel aquaporin-7 gene is altered in severe obesity but not in type 2 diabetes. J Clin Endocrinol Metab (2007) 92:3640–5. 10.1210/jc.2007-0531 17566090

[26] Méndez-Giménez L Becerril S Moncada R Valentí V Ramírez B Lancha A Sleeve gastrectomy reduces hepatic steatosis by improving the coordinated regulation of aquaglyceroporins in adipose tissue and liver in obese rats. Obes Surg (2015) 25:1723–34. 10.1007/s11695-015-1612-z 25736229

[27] Calamita G Gena P Ferri D Rosito A Rojek A Nielsen S Biophysical assessment of aquaporin-9 as principal facilitative pathway in mouse liver import of glucogenetic glycerol. Biol Cell (2012) 104:342–51. 10.1111/boc.201100061 22316404

[28] Jelen S Wacker S Aponte-Santamaria C Skott M Rojek A Johanson U Aquaporin-9 protein is the primary route of hepatocyte glycerol uptake for glycerol gluconeogenesis in mice. J Biol Chem (2011) 286:44319–25. 10.1074/jbc.M111.297002 22081610 PMC3247941

[29] Elkjaer M Vajda Z Nejsum LN Kwon T Jensen UB Amiry-Moghaddam M Immunolocalization of AQP9 in liver, epididymis, testis, spleen, and brain. Biochem Biophys Res Commun (2000) 276:1118–28. 10.1006/bbrc.2000.3505 11027599

[30] Nicchia GP Frigeri A Nico B Ribatti D Svelto M. Tissue distribution and membrane localization of aquaporin-9 water channel: evidence for sex-linked differences in liver. J Histochem Cytochem (2001) 49:1547–56. 10.1177/002215540104901208 11724902

[31] Lebeck J Gena P O’Neill H Skowronski MT Lund S Calamita G Estrogen prevents increased hepatic aquaporin-9 expression and glycerol uptake during starvation. Am J Physiol Gastrointest Liver Physiol (2012) 302:G365–74. 10.1152/ajpgi.00437.2011 22114114

[32] Li CC Lin EC. Glycerol transport and phosphorylation by rat hepatocytes. J Cell Physiol (1983) 117:230–4. 10.1002/jcp.1041170214 6313704

[33] Hibuse T Maeda N Funahashi T Yamamoto K Nagasawa A Mizunoya W Aquaporin 7 deficiency is associated with development of obesity through activation of adipose glycerol kinase. Proc Natl Acad Sci U S A (2005) 102:10993–8. 10.1073/pnas.0503291102 16009937 PMC1182435

[34] Hara-Chikuma M Sohara E Rai T Ikawa M Okabe M Sasaki S Progressive adipocyte hypertrophy in aquaporin-7-deficient mice: adipocyte glycerol permeability as a novel regulator of fat accumulation. J Biol Chem (2005) 280:15493–6. 10.1074/jbc.C500028200 15746100

[35] Maeda N Funahashi T Hibuse T Nagasawa A Kishida K Kuriyama H Adaptation to fasting by glycerol transport through aquaporin 7 in adipose tissue. Proc Natl Acad Sci U S A (2004) 101:17801–6. 10.1073/pnas.0406230101 15591341 PMC539718

[36] Matsumura K Chang BH Fujimiya M Chen W Kulkarni RN Eguchi Y Aquaporin 7 is a β-cell protein and regulator of intraislet glycerol content and glycerol kinase activity, β-cell mass, and insulin production and secretion. Mol Cell Biol (2007) 27:6026–37. 10.1128/MCB.01525-07 17576812 PMC1952143

[37] Spegel P Chawade A Nielsen S Kjellbom P Rutzler M. Deletion of glycerol channel aquaporin-9 (*Aqp9*) impairs long-term blood glucose control in C57BL/6 leptin receptor-deficient (*db/db*) obese mice. Physiol Rep (2015) 3(9):e12538. 10.14814/phy2.12538 26416971 PMC4600382

[38] Kuriyama H Shimomura I Kishida K Kondo H Furuyama N Nishizawa H Coordinated regulation of fat-specific and liver-specific glycerol channels, aquaporin adipose and aquaporin 9. Diabetes (2002) 51:2915–21. 10.2337/diabetes.51.10.2915 12351427

[39] Carbrey JM Gorelick-Feldman DA Kozono D Praetorius J Nielsen S Agre P. Aquaglyceroporin AQP9: solute permeation and metabolic control of expression in liver. Proc Natl Acad Sci U S A (2003) 100:2945–50. 10.1073/pnas.0437994100 12594337 PMC151446

[40] Lebeck J Cheema MU Skowronski MT Nielsen S Praetorius J Hepatic AQP9 expression in male rats is reduced in response to PPARalpha agonist treatment. Am J Physiol Gastrointest Liver Physiol (2015) 308:G198–205. 10.1152/ajpgi.00407.2013 25477377

[41] Yokoyama Y Iguchi K Usui S Hirano K. AMP-activated protein kinase modulates the gene expression of aquaporin 9 via forkhead box a2. Arch Biochem Biophys (2011) 515:80–8. 10.1016/j.abb.2011.08.002 21867676

[42] Prudente S Flex E Morini E Turchi F Capponi D De Cosmo S A functional variant of the adipocyte glycerol channel aquaporin 7 gene is associated with obesity and related metabolic abnormalities. Diabetes (2007) 56:1468–74. 10.2337/db06-1389 17351148

[43] Kondo H Shimomura I Kishida K Kuriyama H Makino Y Nishizawa H Human aquaporin adipose (AQPap) gene. Genomic structure, promoter analysis and functional mutation. Eur J Biochem (2002) 269:1814–26. 10.1046/j.1432-1033.2002.02821.x 11952783

[44] Sjöholm K Palming J Olofsson LE Gummesson A Svensson PA Lystig TC A microarray search for genes predominantly expressed in human omental adipocytes: adipose tissue as a major production site of serum amyloid A. J Clin Endocrinol Metab (2005) 90:2233–9. 10.1210/jc.2004-1830 15623807

[45] Lebeck J Ostergard T Rojek A Fuchtbauer EM Lund S Nielsen S Gender-specific effect of physical training on AQP7 protein expression in human adipose tissue. Acta Diabetol (2012) 49(Suppl 1):S215–26. 10.1007/s00592-012-0430-1 23001483

[46] Kishida K Kuriyama H Funahashi T Shimomura I Kihara S Ouchi N Aquaporin adipose, a putative glycerol channel in adipocytes. J Biol Chem (2000) 275:20896–902. 10.1074/jbc.M001119200 10777495

[47] Rodríguez A Moreno NR Balaguer I Méndez-Giménez L Becerril S Catalán V Leptin administration restores the altered adipose and hepatic expression of aquaglyceroporins improving the non-alcoholic fatty liver of *ob/ob* mice. Sci Rep (2015) 5:12067. 10.1038/srep12067 26159457 PMC4498231

[48] Fasshauer M Klein J Lossner U Klier M Kralisch S Paschke R Suppression of aquaporin adipose gene expression by isoproterenol, TNFα, and dexamethasone. Horm Metab Res (2003) 35:222–7. 10.1055/s-2003-39478 12778365

[49] Licinio J Negrao AB Mantzoros C Kaklamani V Wong ML Bongiorno PB Sex differences in circulating human leptin pulse amplitude: clinical implications. J Clin Endocrinol Metab (1998) 83:4140–7. 10.1210/jcem.83.11.5291 9814504

[50] Tiniakos DG Vos MB Brunt EM. Nonalcoholic fatty liver disease: pathology and pathogenesis. Annu Rev Pathol (2010) 5:145–71. 10.1146/annurev-pathol-121808-102132 20078219

[51] Chalasani N Younossi Z Lavine JE Diehl AM Brunt EM Cusi K The diagnosis and management of non-alcoholic fatty liver disease: practice guideline by the American Gastroenterological Association, American Association for the Study of Liver Diseases, and American College of Gastroenterology. Gastroenterology (2012) 142:1592–609. 10.1053/j.gastro.2012.04.001 22656328

[52] Falck-Ytter Y Younossi ZM Marchesini G McCullough AJ. Clinical features and natural history of nonalcoholic steatosis syndromes. Semin Liver Dis (2001) 21:17–26. 10.1055/s-2001-12926 11296693

[53] Guerrero R Vega GL Grundy SM Browning JD. Ethnic differences in hepatic steatosis: an insulin resistance paradox? Hepatology (2009) 49:791–801. 10.1002/hep.22726 19105205 PMC2675577

[54] Cai C Wang C Ji W Liu B Kang Y Hu Z Knockdown of hepatic aquaglyceroporin-9 alleviates high fat diet-induced non-alcoholic fatty liver disease in rats. Int Immunopharmacol (2013) 15:550–6. 10.1016/j.intimp.2013.01.020 23415870

[55] Zhang J Zhao Y Xu C Hong Y Lu H Wu J Association between serum free fatty acid levels and nonalcoholic fatty liver disease: a cross-sectional study. Sci Rep (2014) 4:5832. 10.1038/srep05832 25060337 PMC5376058

[56] Calamita G Portincasa P. Present and future therapeutic strategies in non-alcoholic fatty liver disease. Expert Opin Ther Targets (2007) 11:1231–49. 10.1517/14728222.11.9.1231 17845148

[57] Portois L Zhang Y Ladriere L Perret J Louchami K Gaspard N Perturbation of glycerol metabolism in hepatocytes from n3-PUFA-depleted rats. Int J Mol Med (2012) 29:1121–6. 10.3892/ijmm.2012.943 22426780

[58] Miranda M Ceperuelo-Mallafré V Lecube A Hernández C Chacón MR Fort JM Gene expression of paired abdominal adipose AQP7 and liver AQP9 in patients with morbid obesity: relationship with glucose abnormalities. Metabolism (2009) 58:1762–8. 10.1016/j.metabol.2009.06.004 19615702

[59] Gu LY Qiu LW Chen XF Lu L Mei ZC. Oleic acid-induced hepatic steatosis is coupled with downregulation of aquaporin 3 and upregulation of aquaporin 9 via activation of p38 signaling. Horm Metab Res (2015) 47:259–64. 10.1055/s-0034-1384569 25105540

